# Breakthrough Path of Low-Level Equilibrium of China's Policy-Oriented Financing Guarantee Market

**DOI:** 10.3389/fpsyg.2022.918481

**Published:** 2022-06-21

**Authors:** Youqing Lv, Guojian Ma, Juan Ding

**Affiliations:** ^1^School of Management, Jiangsu University, Zhenjiang, China; ^2^School of Economics and Management, Chuzhou University, Chuzhou, China

**Keywords:** policy-oriented financing guarantee, low-level equilibrium, breakthrough path, evolutionary game, China

## Abstract

Policy-oriented financing guarantee schemes are widely adopted in the world to alleviate the financing difficulties of small and medium-sized enterprises. However, the development level of policy-oriented financing guarantee market in China has not reached the desired high-level equilibrium target, even though governments have issued a series of guiding policies. Accordingly, based on the evolutionary game theory, this study establishes and analyzes the game model between local governments, guarantee institutions, and banks. Then, the breakthrough effects of different paths on the low-level equilibrium of the guarantee market are simulated. The results show that strengthening superior government's performance appraisal intensity can only partially delay the “window period” of the low-level equilibrium, while adjusting local governments' compensation coefficients or increasing banks' risk sharing ratio have further synergistic effects on the realization of the high-level equilibrium. Additionally, dynamic reward and penalty mechanism of the local governments can effectively restrain the unbalanced state of financing guarantee market caused by banks' excess compensation risk, and finally impel the stabilization of the high-level equilibrium state.

## Introduction

A high default risk ratio and low credit-ratings of small and medium-sized enterprises (SMEs) is a common issue across both developed and developing economies (Taghizadeh-Hesary et al., 2021). Especially, as the COVID-19 pandemic continues to spread and rebound, the financing pressure of SMEs is further intensified (Ma et al., 2021; Calabrese et al., 2022). To alleviate SMEs' financial constraints, Credit Guarantee Schemes (CGSs) have been widely proposed and used around the world (Cowling et al., 2018; Song et al., 2021).

The policy-oriented financing guarantee industry in China is gradually developing under the guidance of inclusive financial policies. According to the annual reports from 2018 to 2020, the average growth rate of China's financing guarantee market reached about 20%, but the proportion of guarantee balance and financing stock size only reached about 0.24%. In addition, the magnification of guarantee business is only 2–3 times, which is far lower than the 30–50 times of developed countries, while the return on equity of guarantee industry is only about 1%, which is far lower than the 12–14% of banking industry and 6% of non-bank financial institutions (Zhang, 2019). From the perspective of efficiency, China's policy-oriented financing guarantee market shows obvious low-level equilibrium characteristics, such as small magnification, insufficient business volume, and risk-return mismatch (Cui et al., 2011). Therefore, exploring the formation mechanism of low-level equilibrium dilemma and seeking breakthrough paths will have a crucial impact on the SMEs' survival and sustainable development and the healthy development of China's economy and social stability (Su et al., 2022).

In fact, a synthesis of the operating model of the policy-oriented financing guarantee system in most Chinese provinces reveals that the policy-oriented financing guarantee system integrates local governments, guarantee institutions, and banks (Xu et al., 2022). Most notably, the decision theory proposed by Simon (2000) shows that these cooperation subjects related to financing guarantee business have significant psychological characteristics of limited rationality. In other words, faced with the uncertainty of guaranteed compensation, it is difficult for cooperation subjects to reach the high-level equilibrium state through a single cooperation, and they need to improve their own behaviors by imitating and learning optimal strategies (Wang et al., 2016). Furthermore, the lenders and the guarantors bear much more operational and financial risks (Yan et al., 2017), making it difficult to ignore the impact of their risk-averse attitude on the low-level equilibrium of policy-oriented guarantee market. Accordingly, for the purpose of improving the equilibrium level of policy-oriented guarantee market, it is reasonable to explore the adjustment process of the tripartite cooperation strategies and to analyze the internal motivation of the adjustment from the perspective of organizational behavior.

Based on the abovementioned analysis, this study considers local governments, guarantee institutions, and banks in China's policy-oriented financing guarantee market as three different groups, and constructs a tripartite game model through evolutionary game theory. Then the internal evolution processes of their behaviors and the stable conditions of the game system under different equilibrium levels are analyzed. Moreover, this study puts forward decision-making propositions for the development of China's policy-oriented financing guarantee industry, as well as simulate the effective breakthrough paths of low-level equilibrium. The main problems to be solved in this study are as follows:

(1) What are the performance characteristics of low-level equilibrium of China's policy-oriented financing guarantee market?
(2) What are the behavioral evolution regulars of local governments, guarantee institutions, and banks under the different equilibrium levels of China's policy-oriented financing guarantee market?
(3) What are the paths to achieve the high-level equilibrium of China's policy-oriented financing guarantee market? And how to play the synergistic effect of different paths?

Compared with the existing research results, the main contributions are claimed in this study. First, considering the characteristic of efficiency, this study takes the breakthrough of low-level equilibrium as the research starting point, and explores the factors that affect the high-level equilibrium of policy-oriented guarantee market. It theoretically expands the research perspective on the improvement of financing guarantee efficiency. Second, based on China's “government-bank-guarantee” business of policy-oriented financing guarantee, this study builds a tripartite game model of local governments, guarantee institutions, and banks, and interprets the intrinsic behavioral motivation of multi-agent cooperation. The results make up for the lack of research on the regular of organizational behavior in the guarantee market as well as enrich the application of evolutionary game theory. Finally, this study designs practical parameters such as superior government's performance appraisal intensity, compensation coefficient, and risk sharing ratio in the construction of game model. The simulated paths make the research more realistic and provide ideas for the development of China's policy-oriented guarantee industry.

The remainder of this study is structured as follows. The Section Literature Review summarizes the relevant literature. The Section Game Model Construction and Analysis establishes and analyzes the evolutionary game system of local governments, guarantee institutions, and banks. The Section Case and Simulation Analysis provides a case study to simulate and discuss the breakthrough paths of low-level equilibrium of China's policy-oriented financing guarantee market. Finally, the main conclusions and limitations are discussed.

## Literature Review

According to the research purposes of this study, the related literature can be divided into three categories, namely, policy-oriented financing guarantee, the equilibrium of financing guarantee market, and the application of evolutionary game.

### Research on Policy-Oriented Financing Guarantee

Within the framework of game theory and asymmetric information theory, guarantee-related theories such as transaction cost theory of credit financing guarantee, adverse selection and moral hazard theory, and relational loan theory have been formed in the theoretical circle. Stiglitz and Weiss (1981), Chan and Thakor (1987), Berger and Udell (1995), and other economists have made key contributions to the improvement of these theories. On this basis, scholars have carried out in-depth research on the effectiveness, operating mechanism, and risk control of financing guarantee. First, the effectiveness of financing guarantee for SMEs was a controversial topic in the 1990s. Banerjee et al. (1994) and Levitsky (1997) believed that credit guarantee can relieve the risks of lending to SMEs, while Gudger (1997) argued that the existence of credit guarantee system will exacerbate the problem of information asymmetry in credit financing market. With the deepening of financing guarantee practice, scholars tried to measure the external effects of financing guarantee (Oh et al., 2009). For instance, Uesugi et al. (2010), Liang et al. (2017), Cowling et al. (2018), Gropp et al. (2020), and Wang et al. (2020) used statistical or econometric models to test the effectiveness of credit guarantees in South Korea, Italy, Japan, and other countries, and agreed that credit guarantee has significant effects in easing credit constraints, reducing the incidence of credit rationing and improving loan availability. Second, for the operating mechanism, the research results are mainly reflected in the comparison and selection of operating models (Craig et al., 2007; Beck et al., 2010), and the construction of operating systems (Boschi et al., 2014; Halunko et al., 2018), which suggest that financing guarantee business is generally implemented through policy-oriented guarantee (Luo and Huang, 2017). Third, in order to ensure the stable operation of policy-oriented guarantee mechanism, scholars further explored the issue of guarantee risk, including risk assessment of guarantee products (Kim and Ahn, 1997; He and Weng, 2014), identification of risk factors in guarantee networks (Berkman et al., 2009; He et al., 2019), guarantee risk control mechanism (Jeon and Sohn, 2008; Huang et al., 2021).

### Research on the Equilibrium of Financing Guarantee Market

As far as we know about the existing research results, few scholars directly explore the equilibrium of policy-oriented financing guarantee market. Conversely, most scholars study the abovementioned equilibrium problem indirectly from the perspective of efficiency, as efficiency is one of the typical characteristics reflecting the equilibrium state of a market or economic system (Fama, 1991). For instance, Ong et al. (2003) and Cui et al. (2011) found that the supply efficiency of credit guarantee market in Malaysia or China was generally low, while Seo and Park (2013) proved that the operation efficiency in Japanese was relatively high, that is, the average efficiency reached 87.4–93.2%. In order to explain the internal mechanism of the difference in guarantee efficiency between different countries, Ono et al. (2013), Li and Lin (2017), Haas and Millone (2020), and Bachas et al. (2021) proposed that government administrative intervention, multi-agent cooperation, information sharing, and risk compensation are the key factors affecting guarantee efficiency. However, the former research results are mostly based on the methods of financial ratio analysis, regression model analysis, and propensity score matching evaluation (Oh et al., 2009), which tend to ignore the dynamic changes of guarantee market and the interaction of business behaviors between guarantee subjects. Therefore, some scholars explored the operation behavior of financing guarantee institutions (He et al., 2014), financing equilibrium under the effect of credit guarantee (Yan et al., 2016; Wang et al., 2022), and factors influencing equilibrium efficiency (Yan et al., 2017, 2020) within the completely rational traditional game framework.

### Research on the Application of Evolutionary Game

Due to the developments of evolutionarily stable strategy and replicating dynamic equation, evolutionary game theory has become a useful tool to explore the behavioral interaction mechanisms between different participating groups (Hofbauer and Sigmund, 2003; Dindo and Tuinstra, 2011), which the core thought is to achieve the final equilibrium through continuous learning and adjustment in market decision-making (Liu et al., 2017). Meanwhile, unlike traditional games framework, evolutionary game theory is based on bounded rationality and limited information, which is more in line with economic reality (Xu et al., 2022). This advantage makes evolutionary game widely used in the financial field. For instance, Wang et al. (2016) used the stability principle of differential equations to analyze the adjustable range of operation mechanisms such as risk sharing ratio and government risk subsidy when the guaranteed market reached an equilibrium state. Weng and Luo (2021) and Xu and Liu (2021) constructed multi-agents evolutionary game models of bank credit market and P2P lending market, respectively, to verify the positive effect of guaranteed mechanism on the improvement of market equilibrium level. Moreover, evolutionary game theory is also used to solve problems in other fields, such as green building construction incentive (Meng et al., 2021) and pandemic prevention (Zhuang et al., 2021). Therefore, the evolutionary game theory is suitable for describing the behavioral interaction mechanism of multi-agents in China's policy-oriented guarantee system.

Reviewing the existing literatures, it can be found that there are deviations in the operational efficiency and equilibrium state of financing guarantee market in different countries, and the research on the mechanism of improving equilibrium level is relatively limited, especially lacking a clear understanding of the multi-agent behaviors in the low-level equilibrium state. To some extent, the existing theoretical achievements are difficult to effectively guide the practice of China's policy-oriented financing guarantee. In fact, the development of China's policy-oriented guarantee system is a behavioral interaction process of continuous game between local governments, guarantee institutions, and banks (Wang et al., 2016). Accordingly, their behavior will have an important impact on the equilibrium level of policy-oriented guarantee market. In view of the above deficiencies, this study will select evolutionary game method to expand the research on the mechanism of improving the equilibrium level of policy-oriented guarantee market.

## Game Model Construction and Analysis

### Game Scenario

According to the guidance policy issued by the State Council of China (2019), local governments are responsible for supervising and encouraging the development of guarantee business, guarantee institutions focus on the development of SMEs' guarantee business under the premise of sustainable operation, and banks actively seek risk sharing and other business cooperation with guarantee institutions. In summary, local governments, depending on their financial strength, have two strategies, including continuous compensation or non-continuous compensation, denoted as (*A*_1_,*A*_2_). Accordingly, faced with the assessment requirement for maintaining and increasing the value of state-owned capital, guarantee institutions also have two strategies of expand business scale and non-expand business scale. While banks' strategy is to adopt actively risk sharing when the compensatory risk is controllable, otherwise, passively risk sharing is adopted. Similarly, the strategies of guarantee institutions and banks can be denoted as (*B*_1_,*B*_2_) and (*C*_1_,*C*_2_), respectively.

### Basic Assumptions

For the convenience of analysis, the following basic assumptions are given:

Assumption 1: According to the basic nature of the evolutionary game (Smith, 1974), local governments, guarantee institutions, and banks have bounded rationality and take decision-making behaviors based on their own interests. Let *x*, *y*, and represent the probability of strategy *A*_1_, *B*_1_, and *C*_1_, respectively. Correspondingly, 1−*x*, 1−*y*, and 1−*z* represent the probability of strategy *A*_2_, *B*_2_, and *C*_2_, respectively. Thereinto, *x, y, z*∈[0, 1].

Assumption 2: Due to the positive externality of credit guarantee to government economy (Riding et al., 2007), if guarantee institutions adopt strategy *B*_2_, local governments will obtain revenue *R*_1_; Conversely, the revenue will increase to *R*2. Meanwhile, the financial departments of local government have set up a special fund for compensatory loss compensation and business expansion subsidies of guarantee institutions (Institute of Fiscal Science, 2010), hence, letting α and β represent compensatory compensation coefficient and business compensation coefficient, respectively. Furthermore, when local governments adopt strategy *A*_2_, the loss caused by the superior government's performance appraisal is *F*. Thereinto, α, β∈[0, 1] and *R*_2_ > *R*_1_.

Assumption 3: Although the pursuit of revenue should not be the primary goal of guarantee institutions, it is also necessary to pursue appropriate revenues, otherwise they will fail because of continuous losses (Wang et al., 2016). Consequently, assuming that the main revenue of guarantee institutions is the guarantee fee generated by guarantee business (Taghizadeh-Hesary et al., 2021), that is, the guarantee fees of strategy *B*_2_ and strategy *B*_1_ are *R*_3_ and *R*_4_, respectively. However, the existence of information asymmetry in the credit market (Altman et al., 2018; Wellalage and Fernandez, 2019) makes guarantee institutions bear the inevitable compensatory loss, denoted as (*D*_2_,*D*_1_) corresponding to strategy (*B*_1_,*B*_2_). Thereinto, *R*_4_ > *R*_3_ and *D*_2_ > *D*_1_.

Assumption 4: Similar to the revenue of local governments, when guarantee institutions adopt strategy *B*_1_ and strategy *B*_2_, the interest revenue of banks is *R*_6_ and *R*_5_, respectively. The positive strategy *A*_1_ of local governments is also beneficial for banks to obtain revenue *R*_7_ because of reducing business risk. In particular, local governments encourage banks to fulfill their risk sharing responsibilities through preferential tax policies (Zhang, 2019). Certainly, the tax incentive coefficient can be reasonably set as θ. In terms of losses, the multi-agent risk sharing mechanism (Xu et al., 2022) forces banks to bear compensatory loss while adopting positive strategy *C*_1_, that is, the risk sharing coefficient is set as γ. Additionally, the banks' negative strategy will also lead to penalty loss *K* given by local governments. Thereinto, θ, γ∈[0, 1] and *R*_6_ > *R*_5_.

According to the assumptions mentioned above, the payment matrix of the game between local governments, guarantee institutions, and banks is summarized in Table 1.

**Table 1:** The payment matrix of the evolutionary game model.

**Players**				**Local governments**	
				***A*_1_**	***A*_2_**
Guarantee institutions	*B*_1_	Banks	*C*_1_	$\left[\begin{array}{c} R_{2}-\alpha \left(1-\gamma \right)D_{2}-\beta R_{4}\\ \left(1+\beta \right)R_{4}-\left(1-\alpha \right)\left(1-\gamma \right)D_{2}\\ \left(1+\theta \right)R_{6}+R_{7}-\gamma D_{2} \end{array}\right]$	$\left[\begin{array}{c} R_{2}-F\\ R_{4}-\left(1-\gamma \right)D_{2}\\ \left(1+\theta \right)R_{6}-\gamma D_{2} \end{array}\right]$
			*C*_2_	$\left[\begin{array}{c} R_{2}+K-\alpha D_{2}-\beta R_{4}\\ \left(1+\beta \right)R_{4}-\left(1-\alpha \right)D_{2}\\ R_{6}+R_{7}-K \end{array}\right]$	$\left[\begin{array}{c} R_{2}+K-F\\ R_{4}-D_{2}\\ R_{6}-K \end{array}\right]$
	*B*_2_	Banks	*C*_1_	$\left[\begin{array}{c} R_{1}-\alpha \left(1-\gamma \right)D_{1}\\ R_{3}-\left(1-\alpha \right)\left(1-\gamma \right)D_{1}\\ \left(1+\theta \right)R_{5}+R_{7}-\gamma D_{1} \end{array}\right]$	$\left[\begin{array}{c} R_{1}-F\\ R_{3}-\left(1-\gamma \right)D_{1}\\ \left(1+\theta \right)R_{5}-\gamma D_{1} \end{array}\right]$
			*C*_2_	$\left[\begin{array}{c} R_{1}+K-\alpha D_{1}\\ R_{3}-\left(1-\alpha \right)D_{1}\\ R_{5}+R_{7}-K \end{array}\right]$	$\left[\begin{array}{c} R_{1}+K-F\\ R_{3}-D_{1}\\ R_{5}-K \end{array}\right]$

### Game System Stability Analysis

Combined with the Malthusian dynamic equation theorem (Friedman, 1998), the expected utilities of the three players' strategies can be obtained, seen in Appendix A.

Thus, the replicating dynamic equations of local governments, guarantee institutions, and banks can be given in the following formula:


(1) $$\left\{ \begin{array}{l} F(x)=x(1-x)\{yz\alpha \gamma (D_{2}-D_{1})-y[\alpha (D_{2}-D_{1})+\beta R_{4}]\\ \;+z\alpha \gamma D_{1}+F-\alpha D_{1}\}\\ F(y)=y(1-y)\{-xz\alpha \gamma (D_{2}-D_{1})+x[\beta R_{4}+\alpha (D_{2}-D_{1})]\\ \;+z\gamma (D_{2}-D_{1})+R_{4}-R_{3}-D_{2}+D_{1}\}\\ F(z)=z(1-z)\{y[\theta (R_{6}-R_{5})-\gamma (D_{2}-D_{1})]\\ \;+\theta R_{5}+K-\gamma D_{1}\} \end{array}\right.$$

According to Formula (1), the first derivatives of *F*(*x*), *F*(*y*), and *F*(*z*) are shown in the following formula:


(2) $$\left\{ \begin{array}{l} F^{\prime }(x)=(1-2x)\{yz\alpha \gamma (D_{2}-D_{1})-y[\alpha (D_{2}-D_{1})+\beta R_{4}]\\ \;+z\alpha \gamma D_{1}+F-\alpha D_{1}\}\\ F^{\prime }(y)=(1-2y)\{-xz\alpha \gamma (D_{2}-D_{1})+x[\beta R_{4}+\alpha (D_{2}-D_{1})]\\ \;+z\gamma (D_{2}-D_{1})+R_{4}-R_{3}-D_{2}+D_{1}\}\\ F^{\prime }(z)=(1-2z)\{y[\theta (R_{6}-R_{5})-\gamma (D_{2}-D_{1})]\;\\ \;+\theta R_{5}+K-\gamma D_{1}\} \end{array}\right.$$

To simplify the calculation, we set $x_{0}=\frac{z\gamma \left(D_{2}-D_{1}\right)+R_{4}-R_{3}-D_{2}+D_{1}}{z\alpha \gamma \left(D_{2}-D_{1}\right)-\alpha \left(D_{2}-D_{1}\right)-\beta R_{4}}$, $y_{0}=\frac{\gamma D_{1}-\theta R_{5}-K}{\theta \left(R_{6}-R_{5}\right)-\gamma \left(D_{2}-D_{1}\right)}$, and $z_{0}=\frac{y\left[\alpha \left(D_{2}-D_{1}\right)+\beta R_{4}\right]+\alpha D_{1}-F}{y\alpha \gamma \left(D_{2}-D_{1}\right)+\alpha \gamma D_{1}}$. Then, the evolutionary stability of the three players' strategies can be discussed in different situations, as summarized in Table 2. For example, when the situation of *z*_0_ < *z* < 1 is met, *A*_1_ evolves to local governments' stabilization strategy. Similarly, evolutionary stability of the three players' strategies in other situations can be explained as above.

**Table 2:** Evolutionary stability results of three players' strategies.

**Players**	**Situations**	**Calculation results**	**Stable strategies**
Local governments	*z* = *z*_0_	*F*′(*x*)≡0	*A*_1_,*A*_2_
	0 < *z*<*z*_0_	*F*′(0) <0,*F*′(1)>0	*A*_2_
	*z*0 < *z* <1	*F*′(0)>0,*F*′(1) <0	*A*_1_
Guarantee institutions	*x* = *x*0	*F*′(*y*)≡0	*B*_1_,*B*_2_
	0 < *x*<*x*_0_	*F*′(0)>0,*F*′(1) <0	*B*_1_
	*x*0 < *x* <1	*F*′(0) <0,*F*′(1)>0	*B*_2_
Banks	*y* = *y*_0_	*F*′(*z*)≡0	*C*_1_,*C*_2_
	0 < *y*<*y*_0_	*F*′(0) <0,*F*′(1)>0	*C*_2_
		*F*′(0)>0,*F*′(1) <0	*C*_1_

Let Formula (2), *F*(*x*) = 0, *F*(*y*) = 0, and *F*(*z*) = 0, then the game system always has eight fixed equilibrium points, that is, *E*_1_(0, 0, 0), *E*_2_(0, 0, 1), *E*_3_(0, 1, 0), *E*_4_(0, 1, 1), *E*_5_(1, 0, 0), *E*_6_(1, 0, 1), *E*_7_(1, 1, 0), and *E*_8_(1, 1, 1). According to Formula (2), the Jacobian matrix can be given in Formula (3). The eigenvalues of the game system are summarized in Table 3.


(3) $$\begin{array}{ll} J= & \\ & \left[\begin{array}{lll} (1-2x)\left\{\begin{array}{l} yz\alpha \gamma (D_{2}-D_{1})-y[\alpha (D_{2}-D_{1})\\ +\beta R_{4}]+z\alpha \gamma D_{1}+F-\alpha D_{1} \end{array}\right\} & \;x(1-x)\left[\begin{array}{l} z\alpha \gamma (D_{2}-D_{1})-\beta R_{4}\\ -\alpha (D_{2}-D_{1}) \end{array}\right] & x(1-x)\left[y\alpha \gamma (D_{2}-D_{1})+\alpha \gamma D_{1}\right]\\ \;y(1-y)\left[\begin{array}{l} -z\alpha \gamma (D_{2}-D_{1})\\ +\beta R_{4}+\alpha (D_{2}-D_{1}) \end{array}\right] & (1-2y)\left\{\begin{array}{l} -xz\alpha \gamma (D_{2}-D_{1})+x[\beta R_{4}+\alpha (D_{2}-D_{1})]\\ +z\gamma (D_{2}-D_{1})+R_{4}-R_{3}-D_{2}+D_{1} \end{array}\right\} & \;y(1-y)\left\{\begin{array}{l} -x\alpha \gamma (D_{2}-D_{1})\\ +\gamma (D_{2}-D_{1}) \end{array}\right\}\\ \;0 & \;z(1-z)\left[\begin{array}{l} \theta (R_{6}-R_{5})\\ -\gamma (D_{2}-D_{1}) \end{array}\right] & (1-2z)\left\{\begin{array}{l} y[\theta (R_{6}-R_{5})-\gamma (D_{2}\\ -D_{1})]+\theta R_{5}+K-\gamma D_{1} \end{array}\right\} \end{array}\right] \end{array}$$

**Table 3:** The eigenvalues and stability of the game system.

**Equilibrium point**	**Eigenvalues**			**Stability**
	**λ_1_**	**λ_2_**	**λ_3_**	
E_1_(0,0,0)	*F*−α*D*_1_	*R*_4_−*R*_3_−*D*_2_+*D*_1_	θ*R*_5_+*K*−γ*D*_1_	Indefinite
E_2_(0,0,1)	*F*−(1−γ)α*D*_1_	*R*_4_−*R*_3_−(1−γ)(*D*_2_−*D*_1_)	−(θ*R*_5_+*K*−γ*D*_1_)	Instability
E_3_(0,1,0)	*F*−α*D*_2_−β*R*_4_	−(*R*_4_−*R*_3_−*D*_2_+*D*_1_)	θ*R*_6_−γ*D*_2_+*K*	Instability
E_4_(0,1,1)	*F*−(1−γ)α*D*_2_−β*R*_4_	−[*R*_4_−*R*_3_−(1−	−(θ*R*_6_−γ*D*_2_+*K*)	Instability
		γ)(*D*_2_−*D*_1_)]		
E_5_(1,0,0)	−(*F*−α*D*_2_)	(1+β)*R*_4_−*R*_3_−(1−	θ*R*_5_+*K*−γ*D*_1_	Indefinite
		α)(*D*_2_−*D*_1_)		
E_6_(1,0,1)	−[*F*−(1−γ)α*D*_1_]	(1+β)*R*_4_−*R*_3_−(1−	−(θ*R*_5_+*K*−γ*D*_1_)	Instability
		α)(1−γ)(*D*_2_−*D*_1_)		
E_7_(1,1,0)	−(*F*−α*D*_2_−β*R*_4_)	−[(1+β)*R*_4_−*R*_3_−(1−	θ*R*_6_−γ*D*_2_+*K*	Indefinite
		α)(*D*_2_−*D*_1_)]		
E_8_(1,1,1)	−[*F*−(1−γ)α*D*_2_−β*R*_4_]	−[(1+β)*R*_4_−*R*_3_−(1−	−(θ*R*_6_−γ*D*_2_+*K*)	ESS
		α)(1−γ)(*D*_2_−*D*_1_)]		

*The column of “Stability” in the table is the results calculated by taking the ideal stable equilibrium point as an example*.

According to the Lyapunov criterion, when all eigenvalues of Jacobian matrix are negative (λ < 0), the equilibrium point is Evolutionary Stable Strategy (ESS). Table 3 summarizes that there are many factors affecting the evolutionary stability of financing guarantee system, and the mathematical relationship between parameters is complex. The eigenvalues of each equilibrium point may be positive or negative, so each of the above equilibrium points may evolve into a stable equilibrium point. In particular, when *E*_1_(0, 0, 0)-*E*_7_(1, 1, 0) are the stable equilibrium points, at least one of the three players will adopt a negative strategy, that is, local governments will not continue to compensate, or guarantee institutions will not expand the business scale, or banks will not insist on sharing compensatory risk. When *E*_8_(1, 1, 1) is the stable equilibrium point, guarantee institutions actively expand guarantee business scale, and local governments and banks insist on fulfilling the responsibilities of compensation and risk sharing, so as to constantly eliminate low-level phenomena. Therefore, *E*_1_(0, 0, 0)-*E*_7_(1, 1, 0) are all stable equilibrium points of the “low-level state,” while only *E*_8_(1, 1, 1) is the ideal stable equilibrium point of the “high-level state.” Taking the ideal stable equilibrium point as an example, the necessary conditions for the financing guarantee market to evolve into the high-level equilibrium state should be met: *F*−(1−γ)α*D*_2_−β*R*_4_ > 0, (1+β)*R*_4_−*R*_3_−(1−α)(1−γ)(*D*_2_−*D*_1_) > 0 and θ*R*_6_−γ*D*_2_+*K* > 0. In this case, the stability of each equilibrium point is summarized in Table 3.

## Case and Simulation Analysis

### A Case Study of China's Policy-Oriented Financing Guarantee

From the analysis mentioned above, it can be seen that China's policy-oriented financing guarantee market may evolve into a “low-level equilibrium state” or a “high-level equilibrium state.” Therefore, this section visualizes the dynamic behaviors of the three players to intuitively reflect the equilibrium state of China's policy-oriented financing guarantee market. Meanwhile, the breakthrough effects of different paths on the evolution of policy-oriented financing guarantee market from “low-level state” to “high-level state” are analyzed. According to the actual operation situation, the financing guarantee system in Hubei Province was explored and established in 2018, which is representative in the practice of policy-oriented financing guarantee for SMEs in China. In one region of this province, the business target of policy-oriented financing guarantee in 2021 is 80 million, but the actual quota is only 33.9 million. The average guarantee rate, repayment rate, and loan interest rate are 1.5, 4.5, and 3.5%, respectively. So, it can be calculated that R_3_ = 3390 * 1.5%≈ 50, *R*_4_ = 8000*1.5% = 120, *R*_5_ = 3390*4.5%≈152, *R*_6_ = 8000*4.5% = 360, *D*_1_ = 3390*3.5%≈118, *D*_2_ = 8000*3.5% = 280. Meanwhile, in combination with the “4321” guarantee business model of this province and the support policies of local governments, other parameters are set as follows: *F* = 5, *K* = 50, α = 0.1, β = 0.05, θ = 0.3, γ = 0.2.

### Evolution Simulation of Guarantee Market's Low-Level Equilibrium

**Figure 1:**
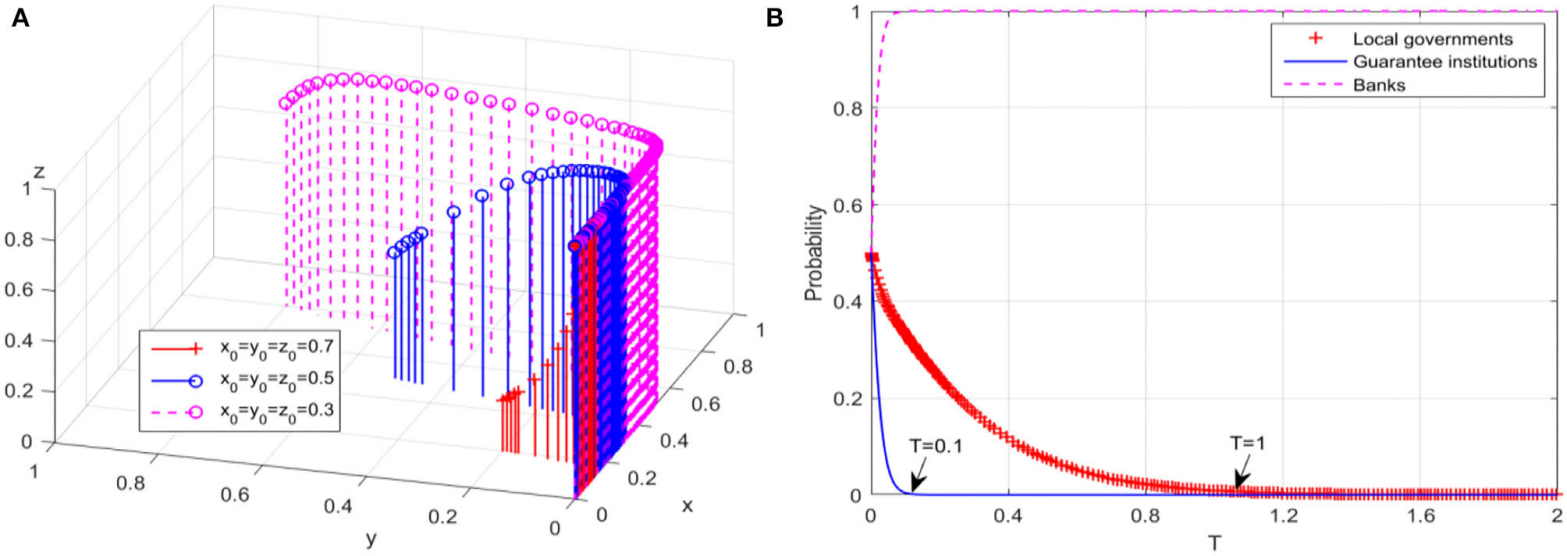
Evolutionary results of low-level equilibrium. **(A)** Game system of guarantee market. **(B)** Behaviors of game players.

The initial willingness of local governments, guarantee institutions, and banks is divided into three levels, namely, high, medium, and low and the values of 0.7, 0.5, and 0.3 are provided, respectively. The simulation results of guarantee market's equilibrium are shown in Figure 1A. It can be found that no matter how the initial interest level changes, *E*_2_(0, 0, 1) evolves into a stable equilibrium point, that is, the guarantee market eventually evolves into a low-level equilibrium state. Taking the initial willingness value of 0.5 as an example, the evolution trajectories of the behavior strategies of the three players under the low-level equilibrium are shown in Figure 1B. Although the probability that banks adopt “actively risk sharing” strategy increases to 1 rapidly, the probability that guarantee institutions adopt “expand business scale” strategy drops to 0 rapidly, and the time inflection point at which local governments' behavior completely evolves into non-continuous compensation is about T = 1. Obviously, there is a “window period” before the guarantee market completely evolves into a low-level equilibrium state, which brings reaction time for the maturity and perfection of the guarantee market.

### Breakthrough Path Simulation of Guarantee Market's Low-Level Equilibrium

As can be seen from the “Evolution simulation of guarantee market's low-level equilibrium section,” during the “window period” of the guarantee market's low-level equilibrium evolution, the behaviors of local governments and guarantee institutions gradually tend to be non-continuous compensation and non-expand business scale, which is exactly opposite to the behavior trajectories of both players under the ideal high-level equilibrium state. In combination with the duplicated dynamic equation (1), parameters such as performance appraisal loss (*F*), compensation coefficients (α, β), risk sharing ratio (γ) have an impact on the behavioral strategies of local governments and guarantee institutions. Therefore, this section simulates the breakthrough effect of the above parameters on financing guarantee market's low-level equilibrium state and the synergistic effect among different parameters.

#### Path 1: Strengthen the Intensity of Performance Appraisal

**Figure 2:**
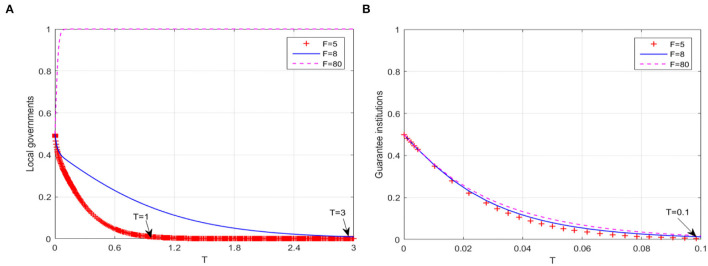
The breakthrough effect of path 1 on low-level equilibrium. **(A)** Local governments **(B)** Guarantee institutions.

In the case of different intensities of local government performance appraisal implemented by superior governments, that is, corresponding loss *F* caused by performance appraisal, the behavioral evolution trajectories of local governments and guarantee institutions are shown in Figures 2A,B, respectively. When *F* increases to 8, there is a significant delay in the complete evolution of local government behavior into non-continuous compensation, that is, the time inflection point is extended from T = 1 to T = 3. And when *F* is large enough, such as increasing to 80, the probability that local governments adopt “continuous compensation” strategy rapidly increases to 1. However, no matter how *F* changes, the behavioral strategy of guarantee institutions does not change, and the delay effect on the time inflection point (T = 0.1) is not significant. Although the game system's stable equilibrium point changes from *E*_2_(0, 0, 1) to *E*_6_(1, 0, 1), the guarantee market is still in the low-level equilibrium state. It can be seen that path 1 can only help to extend the “window period” of local governments' behavior evolution or change its behavior evolution trajectory, but it is difficult to promote the guarantee market to fully reach the high-level equilibrium state. This suggests that synergies of other breakout paths are still needed.

#### Path 2: Adjust the Compensation Coefficients of Local Governments

**Figure 3:**
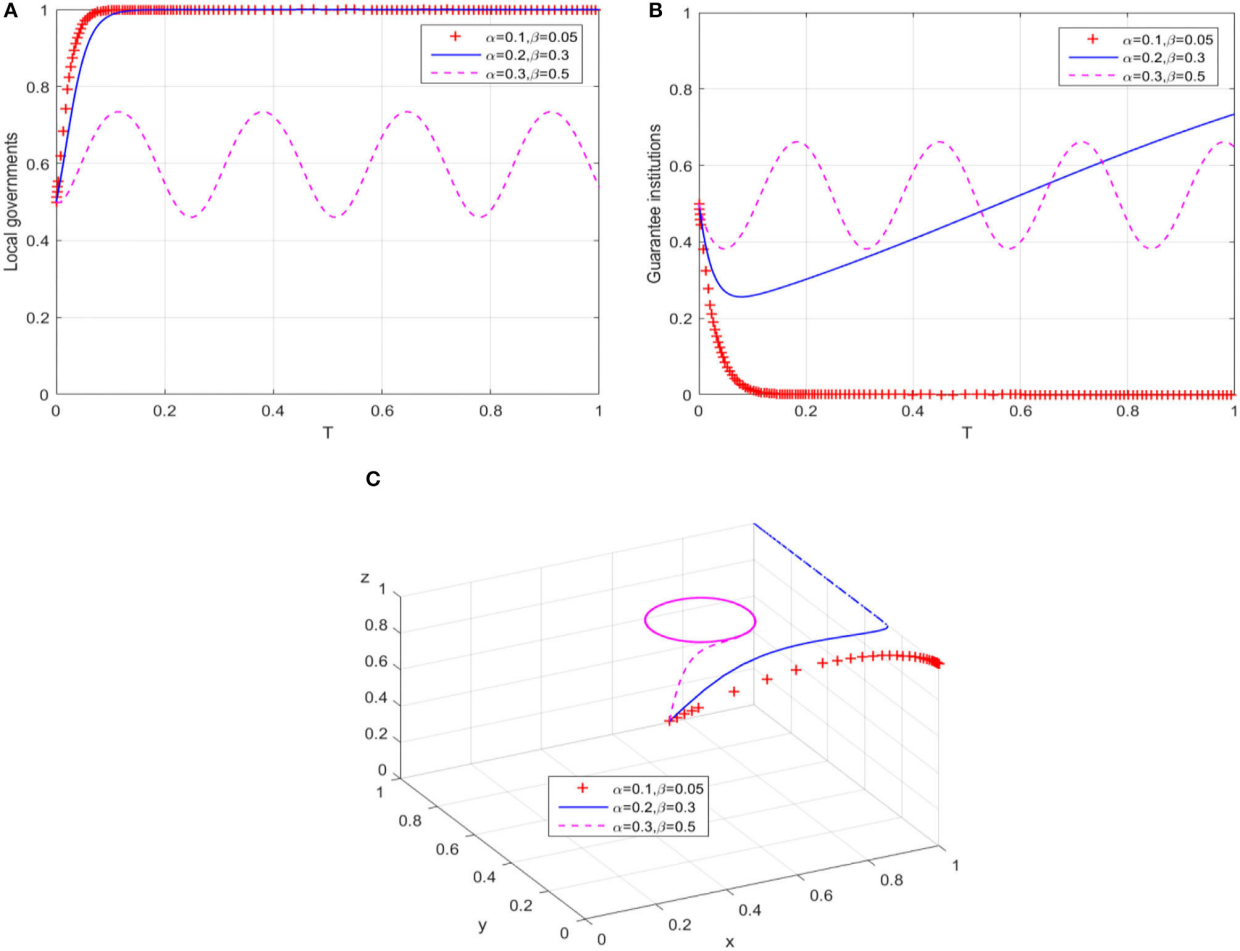
The breakthrough effect of path 2 on low-level equilibrium. **(A)** Local governments **(B)** Guarantee institutions. **(C)** Game system of financing guarantee market.

On the premise that path 1 changes the evolution trajectory of local governments' behavior, that is, *F* = 80, we gradually adjust the compensation coefficients of local governments in path 2. The results of the behavioral evolution trajectories of local governments and guarantee institutions are shown in Figures 3A,B, respectively. When the compensation coefficients are increased to α = 0.2 and β = 0.3 although the time for local governments' behavior to evolve into continuous compensation is delayed, the behavior evolution trajectory of guarantee institutions changes significantly and gradually tends to expand business scale after a short-term shock. Furthermore, when the compensation coefficients continue to increase to α = 0.3 and β = 0.5, both local governments and guarantee institutions show the characteristic of cyclical oscillation, and two players cannot achieve a stable state. From the overall equilibrium evolution of the guarantee market (Figure 3C), the game system's stable equilibrium point evolves from *E*_6_(1, 0, 1) to *E*_8_(1, 1, 1) by regulating local governments to bear more compensatory losses and business expansion subsidies, thus achieving the ideal high-level equilibrium. Nevertheless, when the compensation coefficients reach the critical value, the evolution trajectory of the game system finally transforms into a closed-loop track that oscillates around a certain center point in the plane formed by X-axis and Y-axis. In other words, local governments and guarantee institutions show a cyclical behavior pattern in the game process, and the guarantee market evolves into an unbalanced state.

#### Path 3: Increase Banks' Risk Sharing Ratio

**Figure 4:**
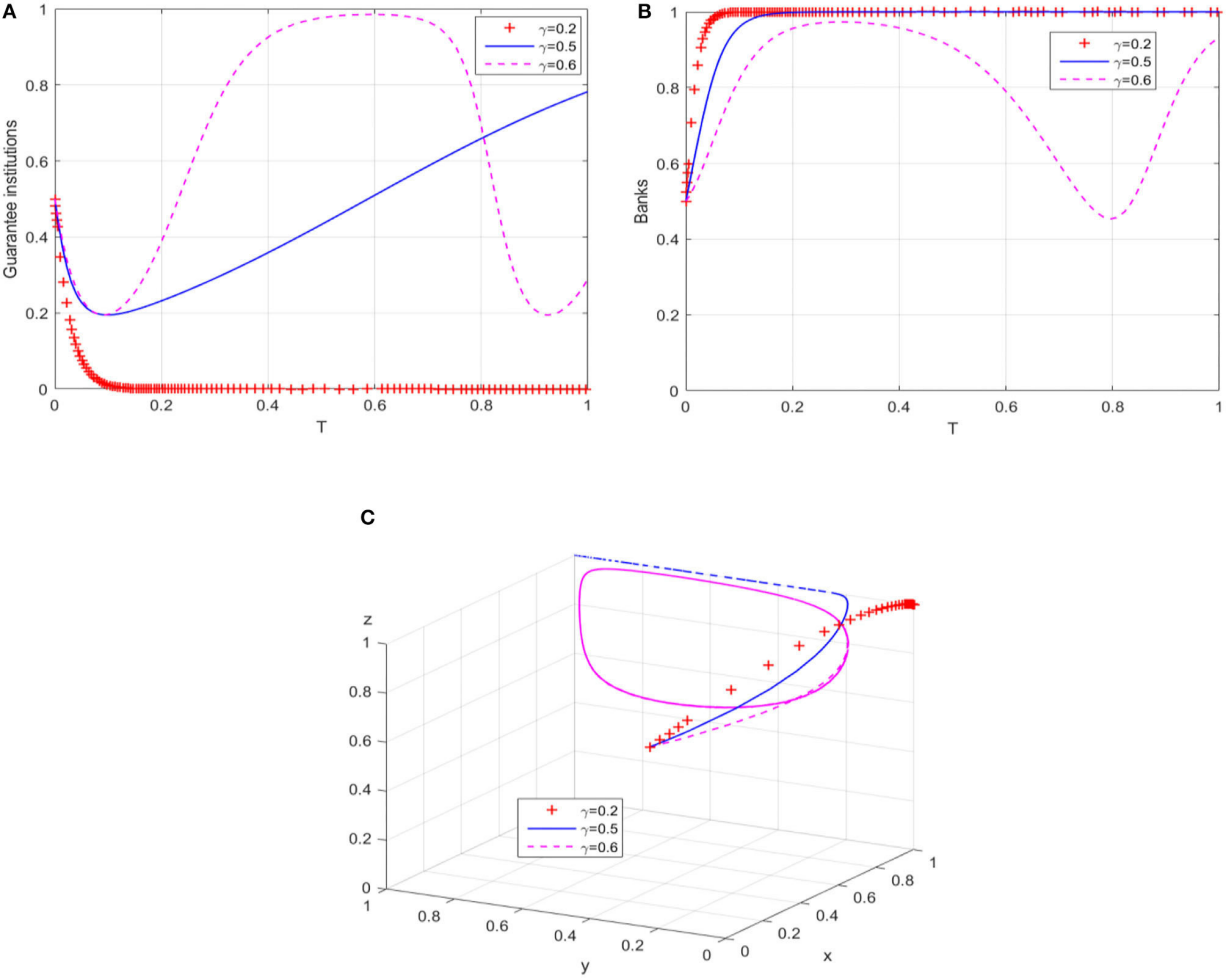
The breakthrough effect of path 3 on low-level equilibrium. **(A)** Guarantee institutions **(B)** Banks. **(C)** Game system of financing guarantee market.

In order to change the behavioral strategy of guarantee institutions in path 1, the ratio is gradually increased from the perspective of banks' risk sharing. The behavioral evolution results of guarantee institutions and banks are shown in Figures 4A,B, respectively. When γ = 0.5, the probability that guarantee institutions adopt an “expand business scale” strategy increases significantly, and the evolution trajectory of banks' behavior does not change significantly; while when γ = 0.6, the behaviors of guarantee institutions and banks also show an unbalanced state of cyclical oscillation. Figure 4C presents the evolution trajectory of the game system under different risk sharing ratios. Within a critical range, increasing banks' risk sharing ratio can effectively promote the evolution of financing guarantee market to the high-level equilibrium. On the contrary, the evolution trajectory of the game system will be transformed into a closed-loop track in the plane formed by Y-axis and Z-axis, which means the cyclical behavior pattern of guarantee institutions and banks in the game process hinders the equilibrium of the guarantee market to a high-level state.

#### Path 4: Implement a Dynamic Reward and Penalty Mechanism

**Figure 5:**
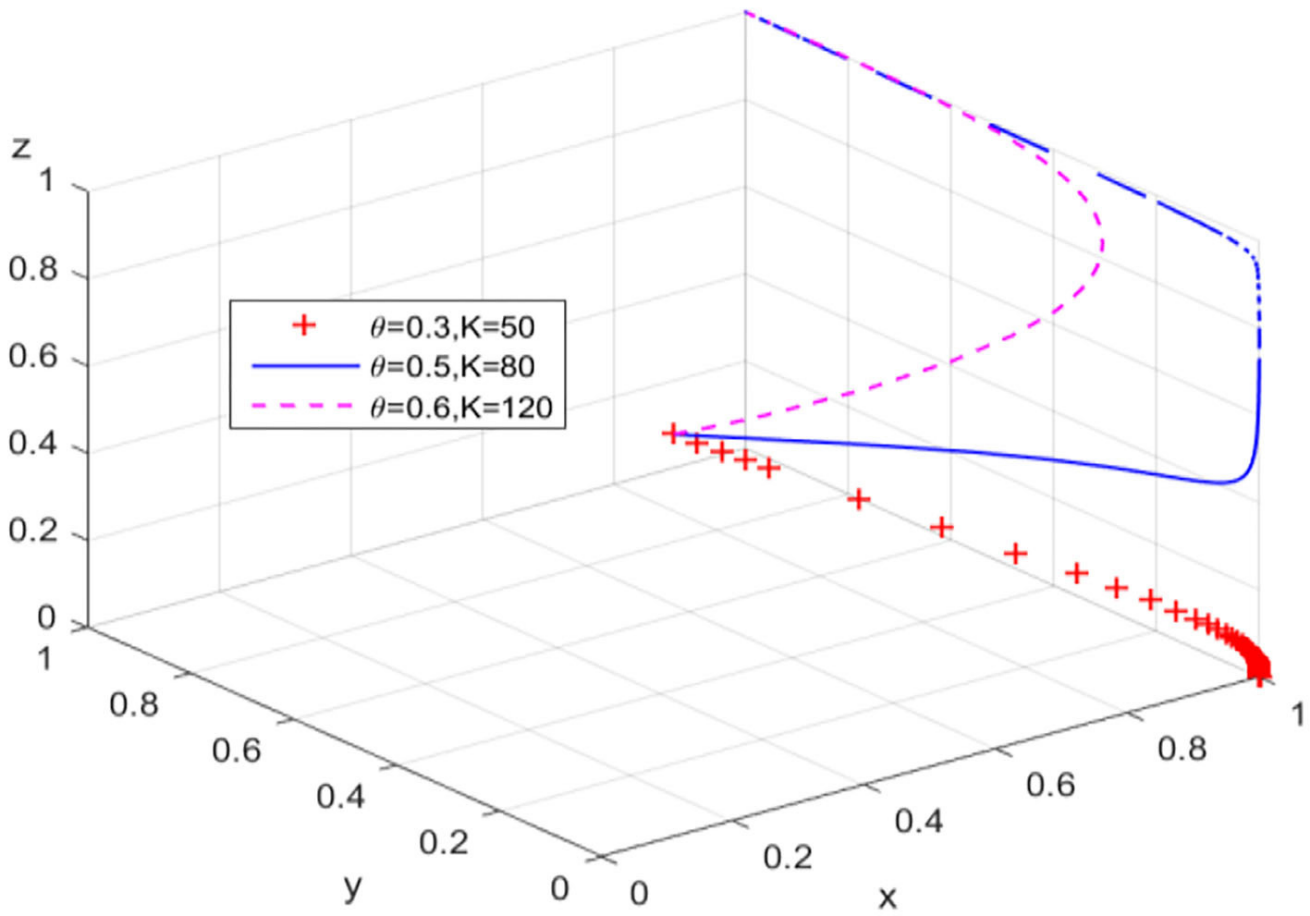
The breakthrough effect of path 4 on low-level equilibrium.

We further discuss the feasibility of the guarantee market evolving to a high-level equilibrium state under high banks' risk sharing ratio (γ = 0.6), and consider a dynamic reward and penalty mechanism of local governments for verification. According to the research methods of Liu et al. (2017) and Meng et al. (2021), it is assumed that local governments' reward and penalty are a linear function of the probability of banks' behavioral strategy. Hence, tax incentive coefficient is θ*z*, and penalty is *K*(1−*z*). The simulation results are shown in Figure 5. It shows that the dynamic reward and penalty mechanism eliminates the cyclical behavior in the game process between guarantee institutions and banks. In addition, with the increase of tax preference coefficient and penalty, the rate of the behavioral probability of both players converging to 1 is also accelerated. Meanwhile, the implementation of dynamic reward and penalty mechanism has no significant impact on local governments' behavioral strategy, and the guarantee market evolves from an unbalanced state to a high-level equilibrium state.

### Results and Discussion

The former simulation results further prove several low-level equilibrium dilemmas that may occur in the policy-oriented financing guarantee market. For instance, Figure 1 shows that when *E*_2_(0, 0, 1) evolves into the stable equilibrium point of the game system, both local governments and guarantee institutions will adopt negative strategies, resulting in low guarantee business volume and unbalanced risk-return market performance, which is in line with the characteristics of low-level equilibrium. Moreover, the result is also consistent with the research of Ong et al. (2003) and Cui et al. (2011), who verified the existence of low-level equilibrium of financing guarantee market in developing countries. The direct reasons for this low-level equilibrium dilemma include, on the one hand, China's relatively high compensation rate makes local governments face greater compensation pressure for a long time, which will be a serious budget burden for local governments with limited financial strength (Institute of Fiscal Science, 2010; Taghizadeh-Hesary et al., 2021). On the other hand, most regions in China currently evaluate guarantee institutions with reference to competitive state-owned enterprises, that is, focusing on the assessment of asset value and profit. This orientation will inevitably strengthen the self-protection awareness of guarantee institutions, and then, they only consider the minimum cost and risk, leading to a large reduction on the scale of guarantee business (Zhang, 2019).

Different from the research of Yan et al. (2016) and Wang et al. (2022), focusing on the realization mechanism of equilibrium state, the simulation results in Figures 2–5 further explain the promotion mechanism of guarantee market's equilibrium level. As we discussed, path 1 simulates the impact of superior government's performance appraisal intensity on the low-level equilibrium state of guarantee market. Although the breakthrough effect is limited, it also corroborates the research of Rong (2014), that is, superior government's performance appraisal is feasible to solve the problem of absence or dislocation of local government functions in the development of market economy. Therefore, path 1 needs to play a synergistic effect with path 2 (local government's compensation mechanism) or path 3 (bank's risk sharing mechanism). Similarly, Wang et al. (2016) also showed the positive effect of government's compensations on the behaviors of guarantee institutions, while Wang (2017) and Huang et al. (2019) demonstrated the positive value of risk sharing on the SMEs financing market. In the case of substantially increasing the banks' risk sharing ratio, path 4 (dynamic reward and penalty mechanism) can effectively eliminate the unbalance of guarantee system, which is consistent with the similar research methods of Liu et al. (2017) and Meng et al. (2021) in other fields. Meanwhile, the suggestion proposed by Xu et al. (2022) that the government should formulate corresponding policy incentive mechanisms enhances the operability of Path 4. In conclusion, “path 1” can guide local governments to the continuous compensation strategy, “path 1 + path 2” or “path 1 + path 3” have a significant positive effect on the willingness of guarantee institutions to adopt expand business scale strategy, and “path 1 + path 3 + path 4” can restrain banks' behavior of passively risk sharing.

## Conclusion and Limitations

To reveal the low-level equilibrium state of China's policy-oriented financing guarantee market, this study constructs an evolutionary game model of local governments, guarantees institutions and banks, and analyzes the complex behaviors of the three game players. Furthermore, the evolution trajectories of the game system under different breakthrough paths are simulated through an operation case, and the boundary conditions for achieving the high-level equilibrium state are discussed. The main research conclusions and related suggestions are as follows.

(1) There is a “window period” before policy-oriented financing guarantee market when evolving into a low-level equilibrium state. However, strengthening superior government's performance appraisal intensity can only change local governments' “non-continuous compensation” behavior in the low-level equilibrium state, which has a limited delay effect on the “window period.” Considering the reality, China's current policy-oriented financing guarantee market is in the growth stage, and the relevant supporting systems are imperfect. Therefore, it is crucial for superior governments to strengthen the supervision over the development of policy-oriented financing guarantee supported by local governments and establish a corresponding assessment mechanism, so as to gain reaction time for the maturity of guarantee market.
(2) Adjusting local government's compensation coefficients or increasing banks' risk sharing ratio, together with strengthening superior government's performance appraisal intensity, can restrain guarantee institutions' “non-expand business scale” behavior in the low-level equilibrium state. Nevertheless, after exceeding a certain critical value, there will be a periodic game behavior between guarantee institutions and local governments or banks, and the guarantee market will evolve from a high-level equilibrium state to an unbalanced state. Consequently, on the one hand, the proportion of local governments' guarantee compensation in the fiscal budget should be increased. On the other hand, banks should be prevented from speculative behaviors of “free-riding” or risk transfer, and the amount of banks' compensation risks should be appropriately raised.
(3) Local governments' dynamic reward and penalty mechanism can effectively restrain the cyclical behavior of banks in the unbalanced state of the guarantee market. Meanwhile, with the increase of reward and penalty coefficient, the unbalanced state of the guarantee market gradually evolves into a high-level equilibrium state. Accordingly, in response to the excess compensation risk borne by banks, it is suggested that local governments design a dynamic tax incentive and penalty mechanism to adjust the interest imbalance of multi-agents, so as to achieve a long-term equilibrium of the high-level state of financing guarantee market.

Meanwhile, this study also has some limitations. The research can be further explored in two aspects in the future. First, the design of a co-governance mechanism with other stakeholders (such as re-guarantee institutions) is worthy of further discussion to improve the equilibrium level of policy-oriented financing guarantee market more effectively. Second, considering the disturbance of external random factors and the complex problems faced by multi-agent cooperation, the stochastic evolutionary game model and the complex network evolutionary game model can describe the improving mechanism of the equilibrium level of policy-oriented guarantee market more effectively in the future research.

## Data Availability Statement

The raw data supporting the conclusions of this article will be made available by the authors, without undue reservation.

## Author Contributions

The first draft of the manuscript was written by YL. The review and editing of the manuscript were performed by GM and JD. All authors contributed to the study conception, material preparation, read, and approved the final manuscript.

## Funding

This research was supported by the key project of National Social Science Foundation of China (17AGL010); Humanities and Social Sciences Research in Colleges and Universities in Anhui Province (SK2018B11); Soft Science Research Project of Jiangsu Province (BR2019026).

## Conflict of Interest

The authors declare that the research was conducted in the absence of any commercial or financial relationships that could be construed as a potential conflict of interest.

## Publisher's Note

All claims expressed in this article are solely those of the authors and do not necessarily represent those of their affiliated organizations, or those of the publisher, the editors and the reviewers. Any product that may be evaluated in this article, or claim that may be made by its manufacturer, is not guaranteed or endorsed by the publisher.

## Appendix A

According to Table 1, the expected utilities of local governments' strategy *A*_1_ and *A*_2_ are as follows:

$$\begin{array}{l} U\left(A_{1}\right)=\left\{\begin{array}{c} yz\left[R_{2}-\alpha \left(1-\gamma \right)D_{2}-\beta R_{4}\right]+\\ y\left(1-z\right)\left[R_{2}+K-\alpha D_{2}-\beta R_{4}\right]+\\ \left(1-y\right)z\left[R_{1}-\alpha \left(1-\gamma \right)D_{1}\right]\\ +\left(1-y\right)\left(1-z\right)\left(R_{1}+K-\alpha D_{1}\right) \end{array}\right\} \end{array}$$

$$\begin{array}{l} U\left(A_{2}\right)=\left\{\begin{array}{c} yz\left(R_{2}-F\right)+y\left(1-z\right)\left(R_{2}+K-F\right)\\ +\left(1-y\right)z\left(R_{1}-F\right)+\\ \left(1-y\right)\left(1-z\right)\left(R_{1}+K-F\right) \end{array}\right\} \end{array}$$

Similarly, the expected utilities of guarantee institutions' strategy *B*_1_ and *B*_2_ are as follows:

$$U(B_{1})=\left\{\begin{array}{l} xz[1+\beta )R_{4}-(1-\alpha )(1-\gamma )D_{2}]\;\\ +x(1-z)[(1+\beta )R_{4}-(1-\alpha )D_{2}]\;\\ \;+(1-x)z[R_{4}-(1-\gamma )D_{2}]\;\\ \;+(1-x)(1-z)(R_{4}-D_{2}) \end{array}\right\}$$

$$\begin{array}{l} U\left(B_{2}\right)=\left\{\begin{array}{c} xz\left[R_{3}-\left(1-\alpha \right)\left(1-\gamma \right)D_{1}\right]\\ +x\left(1-z\right)\left[R_{3}-\left(1-\alpha \right)D_{1}\right]\\ +\left(1-x\right)z\left[R_{3}-\left(1-\gamma \right)D_{1}\right]\\ +\left(1-x\right)\left(1-z\right)\left(R_{3}-D_{1}\right) \end{array}\right\} \end{array}$$

And the expected utilities of banks' strategy *C*_1_ and *C*_2_ are as follows:

$$U(C_{1})=\left\{\begin{array}{c} xy[(1+\theta )R_{6}-\gamma D_{2}]+x(1-y)\\ {}[(1+\theta )R_{5}+R_{7}-\gamma D_{1}]+\\ \left(1-x)y[(1+\theta )R_{6}-\gamma D_{2})]+(1-x)(1-y\right)\\ {}[(1+\theta )R_{5}-\gamma D \end{array}\right\}$$

$$\begin{array}{l} U\left(C_{2}\right)=\left\{\begin{array}{c} xy\left(R_{6}+R_{7}-K\right)+x\left(1-y\right)\left(R_{5}+R_{7}-K\right)+\\ \left(1-x\right)y\left(R_{6}-K\right)+\left(1-x\right)\left(1-y\right)\left(R_{5}-K\right) \end{array}\right\} \end{array}$$

Then, the average expected utilities of local governments, guarantee institutions and banks can be described as follows:

$$\begin{array}{lll} Ū\left(A\right) & = & xU\left(A_{1}\right)+\left(1-x\right)U\left(A_{2}\right)\\ Ū\left(B\right) & = & yU\left(B_{1}\right)+\left(1-y\right)U\left(B_{2}\right)\\ Ū\left(C\right) & = & zU\left(C_{1}\right)+\left(1-z\right)U\left(C_{2}\right). \end{array}$$
